# The cardiovascular risk marker itaconate is sex-dependently associated with legume intake and immune-inflammatory competence in subjects with high BMI

**DOI:** 10.3389/fimmu.2026.1853371

**Published:** 2026-06-02

**Authors:** Amanda Cuevas-Sierra, Andrea Higuera-Gómez, Begoña de Cuevillas, Gabriela Paula-Buestan, María Martínez-Urbistondo, Raquel Castejón, J. Antonio Vargas, José Moisés Laparra, J. Alfredo Martínez

**Affiliations:** 1 Precision Nutrition and Cardiometabolic Health, IMDEA-Nutrition Institute (Madrid Institute for Advanced Studies), Campus of International Excellence (CEI) UAM+CSIC, Madrid, Spain; 2 Department of Pharmacy and Nutrition, Faculty of Biomedical and Health Sciences, Universidad Europea de Madrid-Campus de Villaviciosa de Odón, Madrid, Spain; 3 Internal Medicine Service of Puerta de Hierro, Majadahonda University Hospital, Madrid, Spain; 4 Faculty of Health Sciences, Bioactivity and Nutritional Immunology Group (BIOINUT), Universidad Internacional de Valencia-VIU, Valencia, Spain; 5 Molecular Immunonutrition Group, Madrid Institute for Advanced Studies in Food (IMDEA-Food), Madrid, Spain; 6 Centro de Medicina y Endocrinología, Universidad de Valladolid, Valladolid, Spain; 7 Centro de Investigación Biomédica en Red de La Fisiopatología de La Obesidad y Nutrición (CIBERobn), Instituto de Salud Carlos III, Madrid, Spain

**Keywords:** cardiovascular inflammation, immunometabolism, inflammation, itaconate, legume intake, monocytes, sex differences

## Abstract

**Background:**

Itaconate has received attention as a key immunometabolic mediator produced by activated macrophages, linking cellular metabolism to inflammatory responses. The determinants of circulating itaconate as a cardiovascular risk marker and putative relationships with diet, inflammation, and sex-specific immune responses were assessed given that it remains poorly characterized.

**Objectives:**

The aim of this investigation was to analyze the determinants of circulating itaconate concentrations involving metabolic associations, with markers of innate immune activation and systemic inflammation, as well as to evaluate the potential modulatory role of dietary patterns—particularly legume consumption—as well as sex-specific differences concerning these relationships in subjects with excessive adiposity.

**Methods:**

A total of 453 participants were screened in relation to dietary intake, and also anthropometric measurements, biochemical and inflammatory markers. Participants were categorized according to itaconate levels and legume consumption. Multivariable linear regression models were implemented to identify factors associated with circulating itaconate concentrations. Interaction analyses were performed to assess sex-specific associations.

**Results:**

Circulating itaconate concentrations did not differ across categories of legume intake or adherence to the Mediterranean diet. Baseline characteristics were comparable across groups, with a borderline inverse association with IL-6 levels (p = 0.053). Higher legume consumption was associated with a healthier lifestyle profile and lower adiposity, but not with circulating itaconate. In multivariable analyses, monocyte counts were independently associated with circulating itaconate, while IL-6 showed an independent inverse association. No associations were observed for age, sex, adiposity, dietary variables, or other inflammatory markers. A significant interaction between itaconate and sex was identified for monocyte counts (p = 0.025), with an inverse association observed in men but not in women.

**Conclusions:**

Circulating itaconate appears to reflect innate immune activation rather than dietary exposure *per se*. Association with monocyte-related phenotypes and systemic inflammation, together with marked sex-specific differences, supports a dependent role of itaconate within immunometabolic interactions. The findings highlight notable sex-specific immune responses, underscoring the importance of considering biological sex in understanding variations in immune function in the context of precision medicine.

## Introduction

Cardiovascular disease (CVD) remains the leading cause of morbidity and mortality worldwide and is increasingly recognized as a condition driven partly by chronic low-grade inflammation and dysregulated immune responses (1). Beyond traditional risk factors, the interplay between metabolism and immunity—referred to as immunometabolism—has emerged as a key determinant of cardiovascular health and disease progression (2). Within this framework, metabolic intermediates produced by immune cells are no longer considered passive byproducts, but active regulators of inflammatory pathways (2). Among these metabolites, itaconate has gained considerable attention as a central immunometabolic mediator, which is generated from the tricarboxylic acid (TCA) cycle in activated macrophages through the enzyme encoded by immune-responsive gene 1 (IRG1) (3). Experimental studies have demonstrated that itaconate exerts anti-inflammatory effects by modulating cytokine production, oxidative stress, and key signaling pathways such as Nrf2 and NF-κB (4, 5). In addition, new evidence suggests that itaconate plays a role in cardiovascular pathophysiology, with potential cardioprotective effects mediated through immune and metabolic regulation (6). However, the determinants of circulating itaconate levels in humans, as well as potential relationship with markers of immune activation and systemic inflammation, remain poorly characterized. In this context, circulating inflammatory biomarkers such as interleukin-6 (IL-6) and C-reactive protein (CRP) represent robust indicators of low-grade systemic inflammation and are closely linked to innate immune activation and cardiometabolic risk (ref).

In addition, monocytes and macrophages are central mediators of innate immunity and play a pivotal role in the initiation and progression of cardiovascular inflammation, particularly through their contribution to atherogenesis and plaque destabilization (7, 8). Upon activation, these cells undergo profound metabolic reprogramming, leading to the production of immunoregulatory metabolites such as itaconate via the IRG1 pathway, thereby linking cellular metabolism to inflammatory responses (9). In this context, circulating itaconate levels may serve as a surrogate marker of innate immune activation, as well as a cardiovascular risk marker (10).

Importantly, immune responses are profoundly influenced by biological sex (11). Thus, some studies have demonstrated that men and women exhibit distinct immune phenotypes, including differences in monocyte function, cytokine production, and inflammatory signaling, which contribute to sex-specific susceptibility to cardiovascular and inflammatory diseases (12, 13). However, whether these sex-related differences extend to immunometabolic pathways, including itaconate production and signaling, are scarcely recognized.

Furthermore, dietary factors represent an additional layer of regulation in immunometabolic processes (14). Adherence to healthy dietary patterns, particularly those rich in plant-based foods such as legumes, has consistently associated with reduced inflammation and improved cardiovascular outcomes (15). Legumes are a rich source of dietary fiber, plant protein, and bioactive compounds, including polyphenols and micronutrients, which have been shown to influence systemic inflammatory status and immune function (16). Experimental and clinical studies suggest that these components can modulate immune cell activation and cytokine production, thereby potentially affecting metabolic pathways involved in innate immune responses (17). In this context, legumes represent a relevant dietary component to explore whether dietary exposures may shape circulating immunometabolic and cardiovascular markers such as itaconate, either directly or through the modulation of systemic inflammatory processes. Given their consistent association with anti-inflammatory dietary patterns, legumes may provide a useful proxy to investigate diet-related modulation of immunometabolic responses.

Consequently, the current study aimed to investigate the association between circulating itaconate and markers of innate immune activation, to determine whether dietary factors, particularly legume intake, modulate the interplay between systemic inflammation and circulating itaconate, and to evaluate potential sex-specific differences in these relationships.

## Methodology

### Study design and population

This study is part of the BICAROBESITY-CM (TEC-2024/BIO-307) and METAINFLAMMATION-CM projects (ref. Y2020/BIO-6600), a cross-sectional analysis based on baseline data obtained from participants enrolled in a broader prospective lifestyle intervention cohort. Participants were consecutively recruited from January 2022 at the Internal Medicine Department of Puerta de Hierro Majadahonda University Hospital (Madrid, Spain) and IMDEA Nutrition (Madrid, Spain). A total of 453 adults, including both men and women of Caucasian and Hispanic origin, were included in the study (18). Among them, circulating itaconate concentrations were available for a subset of 182 individuals, who were included in the present analyses. Eight participants were excluded due to outlier circulating itaconate concentrations. The study protocol was approved by the Research Ethics Committee of Puerta de Hierro Majadahonda University Hospital (approval number PI 164–21) and conducted in accordance with the Declaration of Helsinki (19). All participants provided written informed consent prior to enrollment. Inclusion criteria were age >18 years and body mass index (BMI) between 17.0 and 51.4 kg/m². Exclusion criteria included pregnancy or breastfeeding, severe psychiatric disorders, use of medications known to significantly affect body weight, and any condition that could interfere with participation or adherence to study procedures.

### Anthropometric, dietary and clinical measurements

Anthropometric parameters were assessed by trained dietitians following standardized procedures. Body weight and body composition—including total fat mass, visceral fat, and skeletal muscle mass—were measured using a bioelectrical impedance analyzer (TANITA SC-330; Tanita Corp., Tokyo, Japan), following hospital protocols (18). Body mass index (BMI) was calculated as weight (kg) divided by height squared (m²) (20). Dietary intake was assessed using a semi-quantitative food frequency questionnaire (FFQ) (21, 22), in which participants reported habitual consumption frequency for each food item using predefined categories ranging from “never or almost never” to “several times per day,” coded as increasing numerical values.

Legume consumption was derived from the FFQ and categorized into three groups (legumes_cat 1–3) according to increasing frequency of intake, reflecting low, moderate, and high consumption levels. Participants were categorized into three groups according to legume consumption. This categorization approach has been previously used in nutritional epidemiology studies to facilitate interpretation of dietary patterns. Adherence to the Mediterranean dietary pattern was assessed using the validated 14-item Mediterranean Diet Adherence Screener (MEDAS) for the Spanish population (21), as well as physical activity (23).

### Biochemical measurements

Venous blood samples were collected after an overnight fast and analyzed for a range of hematological parameters, including total leukocyte count, lymphocytes, monocytes using an automated hematology analyzer (SYSMEX XN-20; Sysmex Corporation, Kobe, Japan) following validated laboratory procedures. Standard biochemical assays were performed to determine fasting glucose, total cholesterol, glycated hemoglobin (HbA1c), triglycerides and alanine aminotransferase (ALT). Analyses were conducted using a fully automated analyzer (Atellica™ Solution, Siemens Healthineers, Germany) in accordance with the hospital’s standard operating procedures and quality control protocols. Inflammatory and metabolic biomarkers, including C-reactive protein (CRP), fibrinogen, D-dimer, insulin, and interleukin-6 (IL-6), were measured using commercially available enzyme-linked immunosorbent assay (ELISA) kits, following the manufacturers’ instructions.

### Quantification of itaconate

Itaconate was analyzed based on a method described elsewhere (24). Here, it was used a C18 Poroshell 120 column (4.6 x 50 mm; 2.7 μm, Agilent). Aliquots (100 ul) of plasma were mixed with a ACN: MeOH (80:20, v/v) solution, vigorously vortexed (2 min) and centrifuged (6000 xg, 10 min). Samples of clear supernatants (5 ul) were eluted (0.3 ml/min) with 0.2% (v/v) formic acid in water (phase A) and 0.2% (v/v) formic acid in methanol (phase B), using as gradient: 0–6 min, 1% B; 6.0–7.0 min, 90% B; 7.0–8.0 min, 90% B; 8.1–11.0 min, 1% B. Autosampler was set to 10 °C during the analysis. The spectrometer was operated in negative mode. The source was kept configured in an intermediate position, which allowed a balance between sensitivity and stability of the analysis. Scans were recorded over a range of m/z masses between 50 and 2000 atomic mass units. For each scan, the total ion current profile (TIC) was collected, recording the intensity of all ions generated. The signal remained within a variation range of less than 10%, which ensured good reproducibility between runs, although some slight oscillation was to be expected given the ionization conditions used. LOD and lower limit of quantification (LLOQ) of the assay were defined as three times and five times, respectively, the response (peak area) of the matrix blank – LOQ = 25.5 pg/ml and LLOQ = 42.52 pg/ml.

### Statistical analysis

Outliers were defined using the interquartile range (IQR) method, considering values located below Q1 − 1.5 × IQR or above Q3 + 1.5 × IQR, a widely accepted and robust approach for the identification of extreme observations in biomedical data. Following outlier exclusion, the final analytical sample consisted of 174 participants. Participants were categorized into three groups according to legume consumption derived from the semi-quantitative food frequency questionnaire (FFQ) and in two groups of itaconate levels according to the median. In addition, secondary analyses were performed comparing extreme categories of legume consumption (legumes_cat 1 vs. legumes_cat 3) in order to explore diet-dependent associations more clearly. Legume intake was categorized according to weekly consumption frequency as follows: low intake (<2 servings/week), moderate intake (2–4 servings/week), and high intake (>4 servings/week). Continuous variables are presented as mean ± standard deviation (SD), whereas categorical variables are expressed as number of participants and percentages. Differences across the three legume consumption groups were assessed using one-way analysis of variance (ANOVA) for continuous variables and the chi-square test for categorical variables. *Post-hoc* pairwise comparisons were performed using Tukey’s test when appropriate. Linear trends across legume consumption categories were additionally evaluated by trend analysis. For analyses restricted to two groups, differences in continuous variables were assessed using Student’s *t* test, and categorical variables were compared using the chi-square test. Correlation analyses were performed using Spearman’s rank correlation coefficient, and bivariate scatter plots and heatmaps were used for data visualization. To investigate potential effect modification, general linear regression models were fitted including the corresponding interaction terms. Multivariable analyses were adjusted for age and sex when appropriate. Statistical significance was set at *p* < 0.05. All statistical analyses were performed using R software (version 4.3.1; R Foundation for Statistical Computing, Vienna, Austria).

## Results

Baseline characteristics of the study population according to circulating itaconate levels are presented in **Table 1**. Overall, clinical, metabolic, and inflammatory parameters were largely comparable between low and high itaconate groups. No significant differences were observed in age, sex distribution, body mass index, total or visceral adiposity. Markers of systemic inflammation such as C-reactive protein were also similar between groups. However, IL-6 levels showed a borderline difference, with lower levels observed in the high itaconate group (p = 0.053). Dietary factors were also largely comparable between groups. Total legume intake, assessed both as a continuous variable and across consumption categories, did not differ between low and high itaconate groups (p = 0.91 and χ² = 0.036, respectively). Likewise, adherence to the Mediterranean diet, as reflected by the MEDAS score, was similar between groups (p = 0.145).

**Table 1:** Clinical, metabolic, and inflammatory characteristics of the study population according to circulating itaconate levels.

Variable	Low itaconate (n=87)	High itaconate (n=87)	Adjusted p-value
Age (years)	52.14 ± 10.87	53.02 ± 10.61	0.161
Sex, female n (%)	73 (83.9)	65 (74.7)	0.28
Body mass index (kg/m²)	29.08 ± 5.84	29.64 ± 6.21	0.191
Total body fat (%)	36.21 ± 8.92	35.74 ± 9.31	0.192
Visceral fat (AU)	10.10 ± 4.71	10.76 ± 5.77	0.087
Legumes intake Low (<2 serv/d) Medium (3 serv/d) High (>4 serv/d)	16 59 12	17 58 12	0.036
MEDAS score	7.92 ± 1.85	8.34 ± 1.92	0.145
Biochemical data			
Glucose (mg/dL)	94.88 ± 14.20	96.34 ± 17.02	0.302
LDL cholesterol (mg/dL)	112.94 ± 30.41	115.21 ± 31.88	0.355
HDL cholesterol (mg/dL)	57.94 ± 15.68	58.21 ± 16.12	0.771
Triglycerides (mg/dL)	105.33 ± 48.87	109.76 ± 54.11	0.421
Insulin (µU/mL)	10.84 ± 7.12	11.96 ± 8.43	0.278
C-reactive protein (mg/L)	2.31 ± 2.14	2.58 ± 2.31	0.339
IL-6 (pg/mL)	2.77 ± 2.69	1.96 ± 1.51	0.053
Leukocytes (×10³/µL)	6.34 ± 1.92	6.22 ± 1.88	0.593
Monocytes (×10³/µL)	0.41 ± 0.34	0.46 ± 0.52	0.211
Itaconate (μmol/L)	2.19± 4.53	4.87 ± 2.01	<0.001

Data are presented as mean ± standard deviation (SD) or n (%), as appropriate. Comparisons between low and high itaconate groups were performed using Student’s t-test or Mann–Whitney U test for continuous variables, depending on data distribution, and chi-square test for categorical variables. Participants were categorized into low and high circulating itaconate groups according to the median value of circulating itaconate concentrations in the final analytical sample after outlier exclusion (n = 174). Legume intake categories were defined as low (<2 servings/week), moderate (2–4 servings/week), and high (>4 servings/week). AU, arbitrary units; IL-6, interleukin-6. A p-value < 0.05 was considered statistically significant.

Baseline characteristics of the study population according to categories of legume consumption are presented in **Table 2**. Higher legume intake was associated with a more favorable dietary pattern, as reflected by significantly higher adherence to the Mediterranean diet, with increasing MEDAS scores across categories (p < 0.001; p for trend < 0.001). Physical activity also showed a positive trend across legume consumption groups (p for trend = 0.019), although differences did not reach statistical significance (p = 0.062).

**Table 2:** Baseline characteristics of the overall study population according to categories of legume consumption.

Variable	Low intake (n=80)	Medium intake (n=308)	High intake (n=65)	p-value	*Post-hoc* (Tukey)	p for trend
Sex, female n (%)	70 (87.5)	242 (78.6)	46 (70.8)	0.046		
Age (years)	50.61 ± 10.90	54.15 ± 10.60	50.14 ± 11.07	0.003	1 vs 2, 2 vs 3	0.974
Body mass index (kg/m²)	29.14 ± 6.32	29.19 ± 5.32	28.85 ± 6.09	0.904	NS	0.773
Total muscle mass (kg)	46.06 ± 7.97	47.37 ± 9.00	48.94 ± 12.26	0.188	NS	0.068
Total body fat (%)	37.11 ± 9.62	36.03 ± 8.01	32.96 ± 10.26	0.012	1 vs 3, 2 vs 3	0.006
Visceral fat (AU)	9.55 ± 4.80	10.33 ± 4.83	9.68 ± 5.40	0.354	NS	0.788
Legumes (servings/week)	1.73 ± 0.45	3.00 ± 0.00	4.26 ± 0.64	<0.001	all	<0.001
MEDAS score	7.60 ± 1.80	8.02 ± 1.87	9.06 ± 1.85	<0.001	1 vs 3, 2 vs 3	<0.001
Physical activity (METs/week)	890.61 ± 1105.78	1160.91 ± 1226.09	1364.88 ± 1361.88	0.062	NS	0.019
Biochemical data						
Glucose (mg/dL)	93.39 ± 12.52	95.30 ± 16.06	96.90 ± 18.40	0.426	NS	0.193
Total cholesterol (mg/dL)	188.47 ± 35.11	193.60 ± 33.22	194.95 ± 38.77	0.448	NS	0.248
LDL cholesterol (mg/dL)	111.43 ± 33.13	113.88 ± 29.18	116.80 ± 32.34	0.591	NS	0.306
HDL cholesterol (mg/dL)	56.97 ± 15.18	58.70 ± 16.05	56.90 ± 15.63	0.567	NS	0.935
Triglycerides (mg/dL)	100.45 ± 43.80	108.27 ± 52.49	106.08 ± 55.59	0.495	NS	0.463
Glycated hemoglobin (HbA1c, %)	5.39 ± 0.60	5.44 ± 0.48	5.37 ± 0.52	0.512	NS	0.906
ALT (U/L)	22.47 ± 7.55	23.48 ± 8.67	24.02 ± 15.71	0.622	NS	0.343
Leukocytes (×10³/µL)	6.52 ± 2.10	6.18 ± 1.80	6.26 ± 1.96	0.366	NS	0.367
Lymphocytes (×10³/µL)	1.98 ± 0.65	1.87 ± 0.61	2.33 ± 3.19	0.051	2 vs 3	0.191
Monocytes (×10³/µL)	0.38 ± 0.16	0.43 ± 0.47	0.52 ± 0.86	0.298	NS	0.126
C reactive protein (mg/L)	4.98 ± 9.65	3.61 ± 5.85	2.65 ± 3.34	0.18	NS	0.09
IL-6 (pg/mL)	2.25 ± 1.94	2.77 ± 4.08	2.80 ± 2.39	0.45	NS	0.30
Itaconate (µmol/L)	2.93 ± 3.59	3.94 ± 4.99	3.16 ± 3.65	0.440	NS	0.657

Data are presented as mean ± standard deviation (SD) for continuous variables and number (percentage) for categorical variables. Analyses presented in this table were performed in the overall study cohort (n = 453), independently of circulating itaconate data availability. Differences between groups were assessed using one-way analysis of variance (ANOVA) for continuous variables and chi-square test for categorical variables. *Post-hoc* comparisons were performed using Tukey’s test. P for trend was calculated using linear regression models across ordered categories of legume consumption. Dietary variables are expressed as servings/week. MEDAS, Mediterranean Diet Adherence Screener; METs, metabolic equivalents of task; HbA1c, glycated hemoglobin; ALT, alanine aminotransferase; IL-6, interleukin-6; NS, not significant.

In terms of body composition, participants in the highest legume intake category exhibited a significantly lower total body fat percentage compared to lower intake groups (p = 0.012; p for trend = 0.006), while no significant differences were observed for body mass index, visceral adiposity, or total muscle mass. Age differed significantly across groups (p = 0.003), although no linear trend was detected, and sex distribution also varied (p = 0.046), with a lower proportion of women in the highest legume intake category.

Most cardiometabolic parameters, including glucose, lipid profile, and glycated hemoglobin, were comparable across groups (all p > 0.05). However, some hematological and inflammatory markers showed trends across categories. Platelet count (p for trend = 0.024) and fibrinogen levels (p for trend = 0.024) decreased across increasing legume intake, while D-dimer levels were significantly higher in the highest intake group (p = 0.012; p for trend = 0.024). Lymphocyte counts showed a borderline difference between groups (p = 0.051), with higher values in the highest intake category. Similarly, hemoglobin levels showed a non-significant upward trend (p for trend = 0.060).

Markers of systemic inflammation, including C-reactive protein and IL-6, did not differ significantly across legume consumption categories, although a non-significant decreasing trend was observed for C-reactive protein (p for trend = 0.09). Importantly, circulating itaconate levels did not differ across legume intake categories (p = 0.440).

Sex-stratified baseline characteristics across extreme categories of legume intake are presented in **Table 3**. Circulating itaconate concentrations did not differ across legume intake categories (p for legumes main effect = 0.625), and no interaction between legume intake and sex was observed (p interaction = 0.707). However, within the lowest legume intake category, circulating itaconate levels were higher in women compared to men (p = 0.043), a difference not observed in the highest intake group.

**Table 3:** Sex-stratified baseline characteristics across extreme categories of legume intake in participants with available circulating itaconate measurements after outlier exclusion.

Variable	Men low intake (n=10)	Women low intake (n=57)	P value	Men high intake (n=19)	Women high intake (n=46)	P value	P legumes (main effect)	P sex (main effect)	P interaction (legumes × sex)
Itaconate (µmol/L)	1.17 ± 0.92	3.09 ± 3.70	0.043	2.53 ± 1.53	3.41 ± 4.24	0.464	0.625	0.345	0.707
Age (years)	51.10 ± 11.42	50.54 ± 10.90	0.887	52.37 ± 10.93	49.22 ± 11.12	0.300	0.659	0.364	0.588
Weight (kg)	87.80 ± 17.14	77.46 ± 19.39	0.104	92.16 ± 19.35	74.23 ± 18.06	0.002	0.582	<0.001	0.356
Body mass index (kg/m²)	28.58 ± 4.19	29.22 ± 6.59	0.679	29.90 ± 6.55	28.41 ± 5.92	0.399	0.704	0.628	0.433
Total muscle mass (kg)	59.83 ± 7.53	44.03 ± 5.73	<0.001	62.61 ± 6.74	43.29 ± 9.16	<0.001	0.962	<0.001	0.266
Total body fat (%)	27.49 ± 5.46	38.53 ± 9.29	<0.001	26.86 ± 9.79	35.47 ± 9.44	0.003	0.104	<0.001	0.547
Visceral fat (AU)	13.05 ± 4.96	9.04 ± 4.60	0.034	14.05 ± 6.52	7.88 ± 3.61	<0.001	0.354	<0.001	0.286
MEDAS score	8.20 ± 1.62	7.51 ± 1.82	0.241	9.37 ± 2.01	8.93 ± 1.79	0.420	<0.001	0.171	0.751
Physical activity (METs/week)	1502.85 ± 1265.26	803.14 ± 1062.57	0.124	1749.37 ± 1910.65	1206.07 ± 1043.73	0.254	0.073	0.020	0.767
Biochemical data									
Glucose (mg/dL)	102.70 ± 22.32	92.00 ± 9.88	0.167	102.33 ± 29.12	94.57 ± 10.82	0.285	0.455	0.007	0.659
Total cholesterol (mg/dL)	181.30 ± 44.91	189.54 ± 33.70	0.589	184.94 ± 47.26	199.24 ± 34.26	0.257	0.191	0.140	0.709
LDL cholesterol (mg/dL)	117.50 ± 42.47	110.52 ± 31.81	0.628	112.61 ± 41.76	118.60 ± 27.77	0.582	0.346	0.923	0.374
HDL cholesterol (mg/dL)	40.10 ± 11.43	59.49 ± 14.07	<0.001	47.44 ± 15.45	60.95 ± 14.01	0.003	0.290	<0.001	0.345
Triglycerides (mg/dL)	118.10 ± 37.36	97.82 ± 44.33	0.142	124.17 ± 86.88	98.33 ± 33.35	0.236	0.851	0.027	0.796
Glycated hemoglobin (HbA1c, %)	5.71 ± 1.11	5.34 ± 0.47	0.351	5.73 ± 0.58	5.24 ± 0.44	0.009	0.431	<0.001	0.642
ALT (U/L)	29.40 ± 6.42	21.43 ± 7.18	0.003	24.22 ± 4.89	23.93 ± 18.58	0.925	0.641	0.180	0.141
Leukocytes (×10³/µL)	6.29 ± 1.96	6.55 ± 2.13	0.704	5.88 ± 1.80	6.42 ± 2.03	0.319	0.609	0.345	0.758
Lymphocytes (×10³/µL)	1.98 ± 0.48	1.98 ± 0.67	0.961	3.26 ± 5.87	1.95 ± 0.65	0.372	0.546	0.107	0.167
Monocytes (×10³/µL)	0.38 ± 0.10	0.38 ± 0.17	0.924	0.79 ± 1.59	0.41 ± 0.15	0.342	0.319	0.084	0.136
C-reactive protein	3.09 ± 5.56	6.09 ± 10.70	0.04	2.34 ± 3.09	2.78 ± 3.46	0.28	0.09	0.18	0.28
IL-6 (pg/mL)	1.88 ± 1.25	2.31 ± 2.03	0.15	2.16 ± 1.84	3.02 ± 2.53	0.22	0.21	0.12	0.32

Values are expressed as mean ± standard deviation (SD). Legume intake was categorized according to weekly consumption frequency as follows: low intake (<2 servings/week), moderate intake (2–4 servings/week), and high intake (>4 servings/week), as derived from the food frequency questionnaire (FFQ). Analyses presented in this table were performed in participants with available circulating itaconate measurements after outlier exclusion and complete data availability. Comparisons between sexes within each category were performed using Student’s t-test for continuous variables and chi-square test for categorical variables. Main effects of legume consumption, sex, and their interaction (legumes × sex) were assessed using general linear models (GLM) adjusted for age and sex, when appropriate. METs, metabolic equivalents of task; HbA1c, glycated hemoglobin; ALT, alanine aminotransferase; IL-6, interleukin-6; AU, arbitrary units.

Higher legume consumption was associated with greater adherence to the Mediterranean diet (p for legumes main effect < 0.001). No differences were observed across categories for age or body mass index.

Sex differences were observed across several variables, with men showing higher body weight, muscle mass, visceral fat, uric acid, glucose, triglycerides, glycated hemoglobin, and urea levels (all p for sex < 0.05), while women had higher HDL cholesterol levels (p for sex < 0.001).

Among inflammatory markers, C-reactive protein was higher in women than men within the lowest intake group (p = 0.040), while no differences were observed for IL-6. A borderline trend was observed for monocyte counts (p for sex = 0.084), although this did not reach statistical significance.

### Determinants of circulating itaconate and association with inflammatory markers

**Figure 1:**
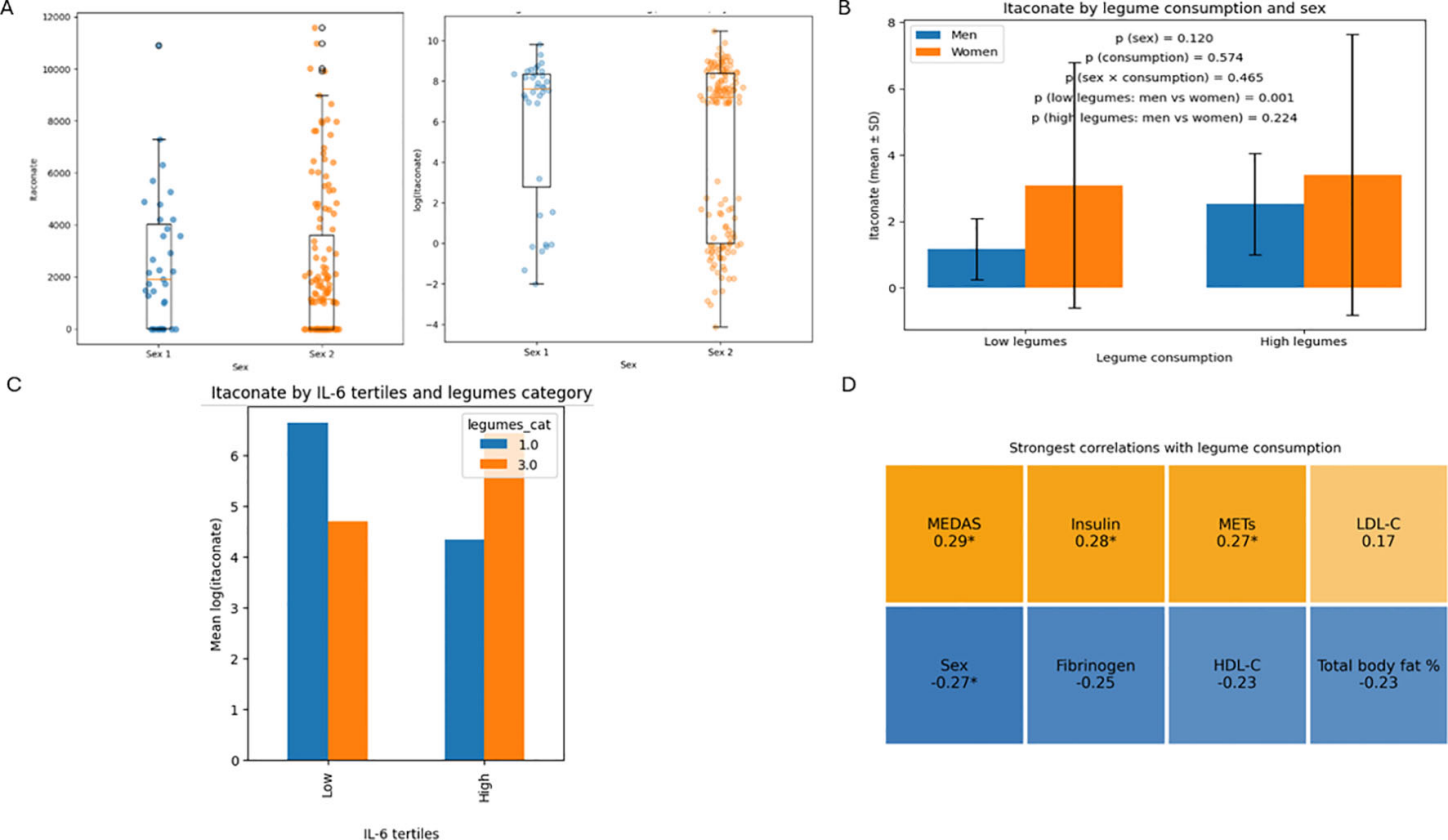
Circulating itaconate levels in relation to sex, legume consumption, inflammatory status, and cardiometabolic profile. **(A)** Distribution of circulating itaconate concentrations by sex, shown as scatter plots with overlaid boxplots for raw (left) and log-transformed (right) values. Each dot represents an individual participant. Boxplots indicate median and interquartile range. Log-transformation was applied to account for the skewed distribution of itaconate concentrations. **(B)** Mean circulating itaconate concentrations according to legume intake (low vs high) and sex. Bars represent mean ± SD. No significant main effects of sex or legume consumption, nor interaction between both factors, were observed. **(C)** Mean log-transformed circulating itaconate concentrations across tertiles of interleukin-6 (IL-6), stratified by legume intake category (low vs high). This panel illustrates potential differences in circulating itaconate levels according to inflammatory status and dietary patterns. **(D)** Heatmap showing the strongest correlations between legume consumption and selected cardiometabolic variables (categories 1 vs 3). Orange indicates positive correlations and blue indicates negative correlations. Color intensity reflects the strength of the association. Asterisks denote statistical significance (*p < 0.05*).

Circulating itaconate concentrations showed a highly skewed distribution, with a substantial proportion of low values, which justified log-transformation for subsequent analyses (**Figure 1A**).

As illustrated in **Figure 1B**, the distribution of raw itaconate levels differed markedly between individuals, whereas log-transformation improved normality and reduced dispersion.

In multivariable analyses, a significant interaction between circulating itaconate and sex was observed in relation to monocyte counts (p for interaction = 0.025). Sex-stratified models revealed an inverse association between circulating itaconate and monocyte counts in men, whereas no significant association was observed in women, suggesting a sex-specific relationship between itaconate and innate immune cell profiles. Circulating itaconate concentrations varied across IL-6 tertiles, with distinct patterns observed according to legume intake category (**Figure 1C**). In individuals with low legume consumption, itaconate levels appeared higher in the lowest IL-6 tertile and decreased in the intermediate tertile, whereas a different pattern was observed among those with higher legume intake. In addition, panel 1D shows the strongest correlations between legume consumption and selected cardiometabolic variables. Legume intake was positively associated with adherence to the Mediterranean diet (MEDAS), insulin levels, and physical activity (METs), while inverse correlations were observed with sex, fibrinogen, HDL cholesterol, and total body fat percentage.

**Figure 2:**
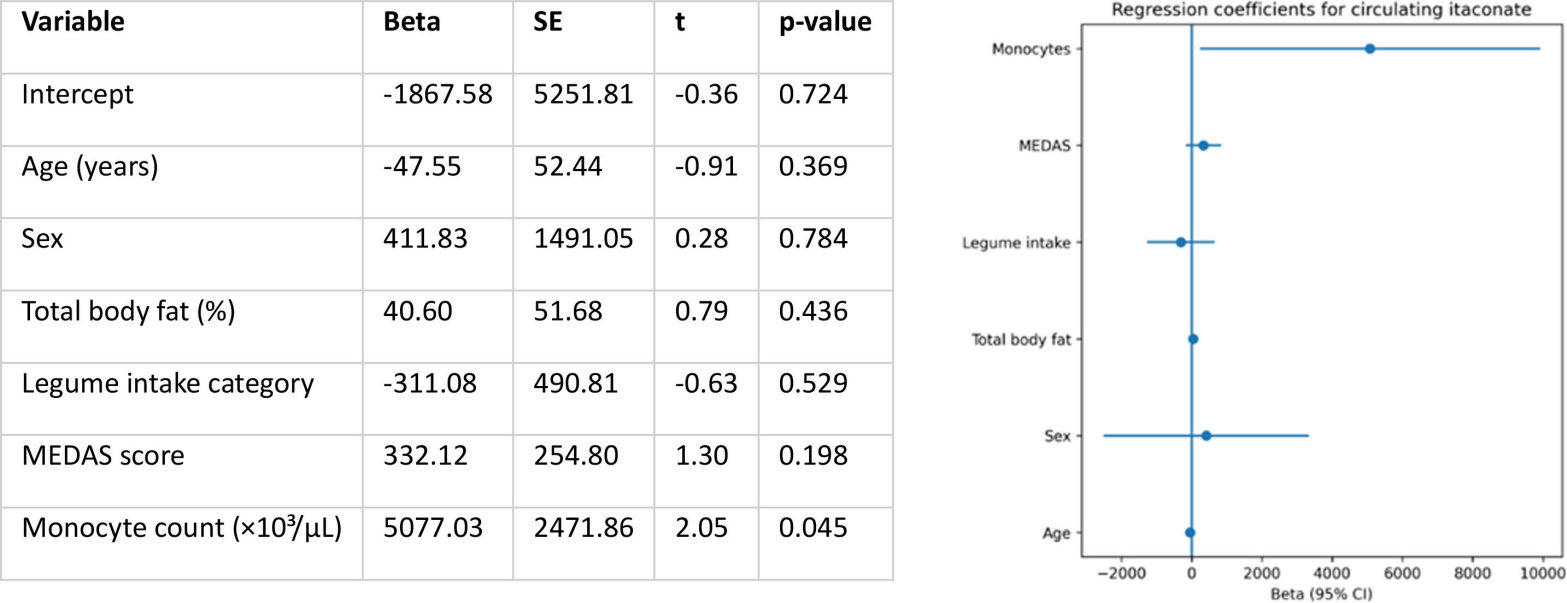
Multivariable linear regression analysis of factors associated with circulating itaconate levels. Points represent beta coefficients and horizontal lines indicate 95% confidence intervals. The vertical line indicates the null value (β = 0). Monocyte count was the only variable significantly associated with circulating itaconate levels. The table represents the multivariable linear regression model evaluating determinants of circulating itaconate concentrations. β values correspond to unstandardized regression coefficients. The model included age, sex, total body fat percentage, legume intake category, Mediterranean diet adherence score (MEDAS), and monocyte counts as independent variables. SE: standard error.

To explore factors associated with circulating itaconate concentrations, multivariable linear regression analyses were performed. After adjustment for age, sex, body fat percentage, legume intake category, and adherence to the Mediterranean diet, monocyte counts were independently associated with circulating itaconate concentrations (β = 5077.03, p = 0.045), whereas no significant associations were observed for age, sex, body fat percentage, legume intake category, or MEDAS score. The model explained a modest proportion of the variance in circulating itaconate concentrations (R² = 0.14; adjusted R² = 0.039), suggesting that additional factors may contribute to inter-individual variability in circulating itaconate levels (**Figure 2**).

**Figure 3:**
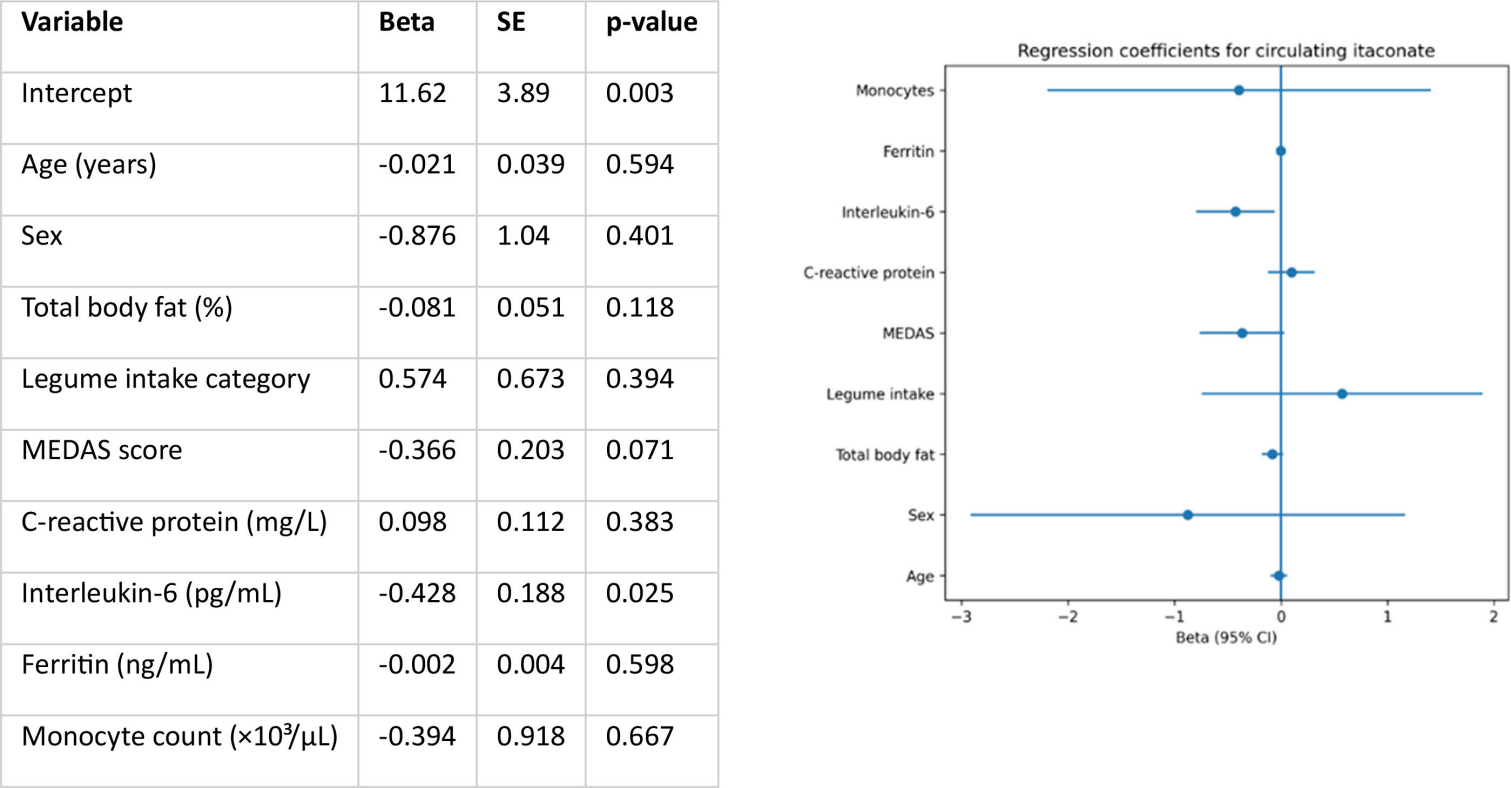
Multivariable linear regression analysis of factors associated with circulating itaconate levels. Points represent beta coefficients and horizontal lines indicate 95% confidence intervals. The vertical line indicates the null value (β = 0). Interleukin-6 was the only variable significantly associated with circulating itaconate levels. The table represents the multivariable linear regression model evaluating factors associated with circulating itaconate concentrations. β values represent unstandardized regression coefficients. The model included age, sex, total body fat percentage, legume intake category, Mediterranean diet adherence score (MEDAS), C-reactive protein (CRP), interleukin-6 (IL-6), ferritin, and monocyte counts as independent variables. SE, standard error.

To further investigate potential links between circulating itaconate and inflammatory pathways, an additional multivariable linear regression model including inflammatory markers was constructed. In this model, circulating interleukin-6 (IL-6) concentrations were independently and inversely associated with circulating itaconate levels (β = −0.428, p = 0.025), whereas C-reactive protein (CRP), ferritin, and monocyte counts were not independently associated (**Figure 3**).

The model explained a modest proportion of the variance in circulating itaconate concentrations (R² = 0.115; adjusted R² = 0.052), suggesting that systemic inflammatory markers, particularly IL-6, may partially contribute to inter-individual variability in circulating itaconate levels.

Scatter plot showing the relationship between log-transformed circulating itaconate concentrations and monocyte counts (×10³/µL), stratified by sex. Regression lines represent predicted values derived from multivariable linear regression models including log(itaconate), sex, the interaction term log(itaconate) × sex, age, body mass index, and legume intake category. A significant interaction between circulating itaconate and sex was observed (p for interaction = 0.025), indicating that the association between circulating itaconate and monocyte counts differs by sex. Men are shown in blue and women in orange.

In multivariable linear regression models additionally adjusted for legume intake category, circulating log-transformed itaconate remained significantly associated with monocyte counts (β = −0.142, p = 0.014). A significant interaction between itaconate and sex was observed (β for interaction = 0.069, p = 0.025), indicating that the association between circulating itaconate and monocyte counts differs by sex. Legume intake category was not independently associated with monocyte counts.

**Figure 4:**
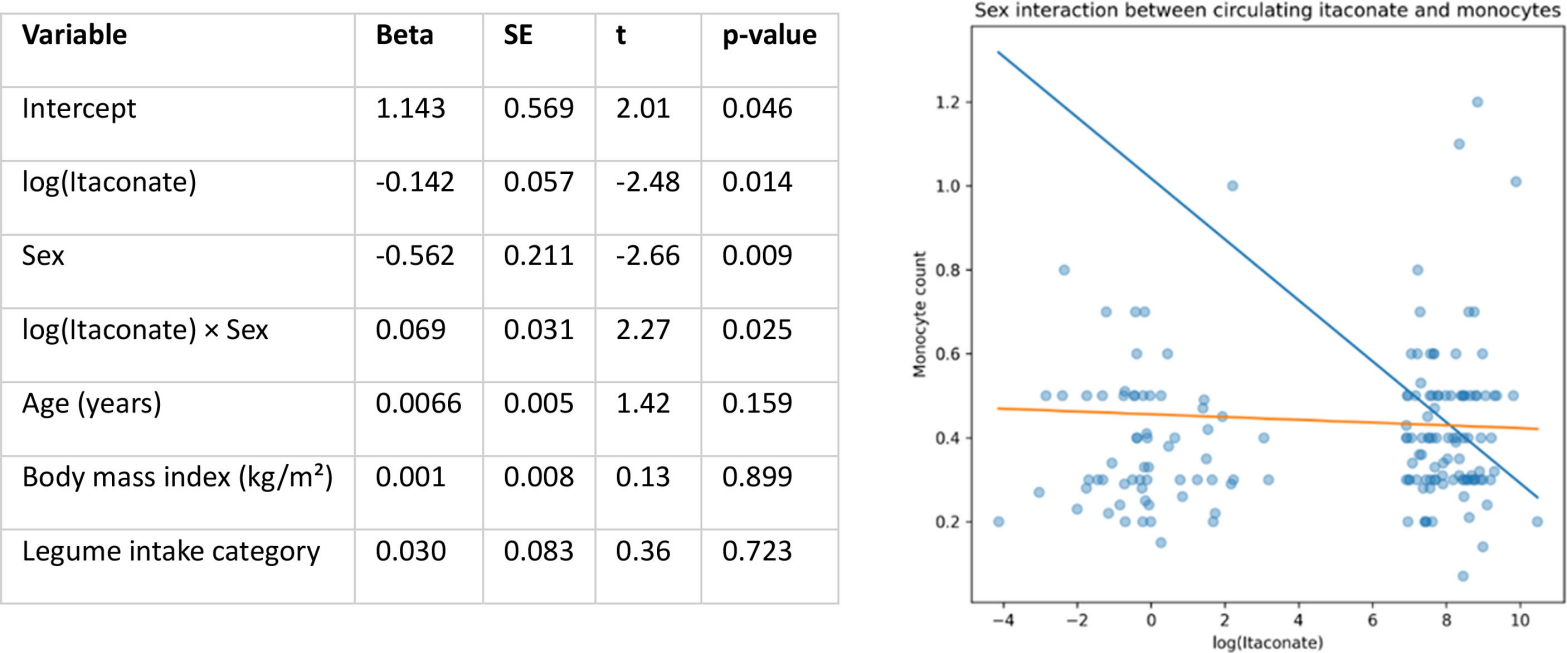
Sex-specific association between circulating itaconate and monocyte counts. Scatter plot showing the relationship between log-transformed circulating itaconate concentrations and monocyte counts (×10^3^/µL), stratified by sex. Regression lines represent predicted values derived from multivariable linear regression models including log(itaconate), sex, the interaction term log(itaconate) × sex, age, body mass index, and legume intake category. A significant interaction between circulating itaconate and sex was observed (p for interaction = 0.025), indicating that the association between circulating itaconate and monocyte counts differs by sex. Men are shown in blue and women in orange. The table represents the multivariable linear regression model evaluating the sex-specific association between circulating itaconate and monocyte counts. β coefficients represent unstandardized regression coefficients derived from multivariable linear regression analyses. The model included log-transformed circulating itaconate concentrations, sex, the interaction term between log(itaconate) and sex, age, body mass index (BMI), and legume intake category as independent variables. Models were adjusted for all listed covariates. SE, standard error.

As illustrated in **Figure 4**, the association between circulating log-transformed itaconate concentrations and monocyte counts differed between men and women. In men, higher itaconate concentrations were associated with lower monocyte counts, whereas this relationship was attenuated in women, supporting a sex-specific association between circulating itaconate and innate immune cell profiles.

## Discussion

The present investigation identifies circulating itaconate as a metabolite linked to innate immune activity in a sex-dependent manner and places this association within a broader dietary and inflammatory context. First, circulating itaconate was associated with monocyte-related immune phenotypes, supporting the view that this metabolite reflects myeloid immunometabolic activation. Second, the relationship between itaconate and monocyte counts differed by sex, suggesting that biological sex modifies the link between innate immune activation and itaconate biology. Taken together, these data support a model in which circulating itaconate is better understood as a context-dependent immunometabolic signal than as a simple readout of dietary exposure.

This interpretation is consistent with the established role of monocytes and macrophages as major drivers of cardiovascular inflammation and as the main cellular source of itaconate (25). In atherosclerosis and related cardiometabolic states, monocyte recruitment, macrophage differentiation, and defective resolution of inflammation are key determinants of plaque evolution and tissue injury (26). Activated macrophages undergo marked metabolic rewiring, and one of the most characteristic features of this process is induction of the ACOD1/IRG1–itaconate axis, which diverts cis-aconitate from the tricarboxylic acid cycle toward itaconate production (9, 27). In that sense, itaconate is not merely a downstream metabolite but part of the metabolic program of activated myeloid cells. Our observation that circulating itaconate tracks with monocyte-associated phenotypes is therefore consistent with the notion that systemic itaconate may partly mirror the activation state, abundance, or functional polarization of innate immune cells participating in cardiovascular inflammation.

A key feature of itaconate biology is that the production is induced in inflammatory macrophages, whereas many of its downstream effects are anti-inflammatory (28). Rather than representing a paradox, this reflects a feedback mechanism whereby inflammatory activation triggers the production of a metabolite that subsequently limits excessive immune responses. Itaconate has been shown to modulate cytokine production, redox balance, and inflammasome-related pathways, positioning it at the interface between inflammatory activation and its resolution (29). In this context, our findings suggest that circulating itaconate may not simply reflect a protective state, but rather the activation of a compensatory immunometabolic program in response to inflammatory stimuli.

The anti-inflammatory effects of itaconate are also tightly linked to macrophage metabolic state. The ACOD1/itaconate pathway emerges during the broader remodeling of central carbon metabolism that accompanies macrophage activation, and in turn feeds back on mitochondrial metabolism, redox balance, and inflammatory signal transduction. This makes itaconate especially relevant in cardiometabolic disease, where nutrient excess, adiposity, insulin resistance, and low-grade inflammation converge on monocyte-macrophage dysfunction (30–32). In our cohort, higher legume intake was associated with lower body fat percentage and higher adherence to a Mediterranean dietary pattern, but not with higher circulating itaconate per se. This lack of a direct dietary association may suggest that in humans, circulating itaconate is unlikely to be determined by one food group alone and is more likely shaped by the overall inflammatory-metabolic context in which myeloid cells operate.

Our finding that IL-6 was independently associated with circulating itaconate further supports a bidirectional relationship between inflammation and itaconate biology. In one direction, inflammatory stimuli induce ACOD1 expression and itaconate synthesis in myeloid cells. In the other, itaconate can reprogram the inflammatory response, thereby shaping the duration, magnitude, and quality of macrophage activation. This reciprocal structure may explain why the association between systemic inflammatory markers and circulating itaconate is not necessarily linear or uniform across individuals. Indeed, the exploratory analyses suggested that the pattern linking IL-6 and itaconate differed according to legume intake, with a more coordinated increase in itaconate across inflammatory strata among those with higher legume consumption. Although this interaction should be interpreted cautiously, it is compatible with the idea that diet does not directly determine circulating itaconate, but can modify the inflammatory context in which the ACOD1/itaconate axis is engaged (32–35).

A particularly relevant result of the present study is the sex-specific association between circulating itaconate and monocyte counts (35). Sex differences in immune function are robust across populations and influence susceptibility to infection, autoimmunity, cancer, and chronic inflammatory disease (36). These differences arise from the combined action of sex chromosomes, steroid hormones, cell-intrinsic signaling programs, and sex-dependent environmental exposures, including microbiome-related effects (37). In the cardiovascular field, sex shapes inflammatory phenotypes, disease presentation, and response to injury (38–41). Against this background, our observation that the itaconate–monocyte relationship was stronger in men, whereas it was attenuated in women, suggests that the same degree of circulating itaconate may not carry identical biological meaning across sexes (42–44). It is plausible that hormonal regulation, differences in monocyte programming, or sex-specific thresholds for inflammatory-metabolic adaptation influence either itaconate production by macrophages or the systemic interpretation of that signal (45–47). The role of sex hormones deserves particular consideration. Steroid hormones regulate immune cell differentiation, cytokine responses, and cellular metabolism, and macrophages are highly responsive to these signals. Estrogens, progesterone, and androgens can alter macrophage activation thresholds, oxidative metabolism, and inflammatory polarization, raising the possibility that they also influence ACOD1 induction, intracellular itaconate handling, or the export and systemic detectability of itaconate (48–50).

An additional layer of complexity arises from the interplay between immunometabolism and epigenetic regulation. Macrophage activation is influenced not only by metabolic rewiring but also by chromatin and transcriptional programs, and itaconate has been implicated in this crosstalk (51, 52). Although not assessed in the present study, this may partly explain why circulating itaconate reflects more than a transient inflammatory signal and likely depends on cellular and disease context.

This context dependence is highly relevant for disease pathophysiology. In cardiovascular disease, recent work supports a role for itaconate in cardiovascular immunometabolism and, in some experimental settings, suggests cardioprotective effects linked to inflammatory restraint (10, 53). At the same time, the biology of itaconate is unlikely to be uniformly beneficial across diseases. In obesity and overnutrition, ACOD1-dependent itaconate production has been implicated in microbiota changes that promote metabolic dysfunction in mice, underscoring that the impact of this pathway depends on nutritional context and host-microbial interactions (54). Likewise, in cancer, macrophage-derived itaconate may foster immune evasion under some conditions (52, 55). Therefore, circulating itaconate should probably not be conceptualized as simply “good” or “bad,” but rather as a dynamic node integrating inflammatory cues, metabolic stress, cellular adaptation, and tissue context.

The possible contribution of diet deserves further attention. In our dataset, higher legume intake clustered with greater intake of vegetables, nuts, fatty fish, olive oil, and a higher MEDAS score, consistent with a broader Mediterranean-like pattern rather than an isolated food effect. This is important because plant-rich diets may influence myeloid immunometabolism indirectly through adiposity, insulin sensitivity, inflammatory tone, microbial fermentation, and the generation of microbiota-derived metabolites (56, 57). At the same time, recent work indicates that the ACOD1/itaconate pathway can itself shape gut microbial ecology in the setting of overnutrition (58). A reasonable interpretation, therefore, is that diet–microbiota–myeloid interactions may influence circulating itaconate in a distributed, systems-level manner that is unlikely to be captured by one dietary variable alone. This may explain why legume intake was not directly associated with itaconate concentration, yet still appeared relevant as a contextual modifier.

Taken together, our results support an integrative conceptual model in which circulating itaconate sits at the interface of four domains: innate immune activation, systemic inflammatory tone, metabolic state, and sex-dependent regulation. In this model, inflammatory and metabolic stressors induce ACOD1-dependent itaconate production in myeloid cells; circulating itaconate then reflects, at least in part, the magnitude and quality of this activation (59). Dietary exposures can modulate the baseline inflammatory-metabolic environment in which this pathway operates, while sex hormones and sex-linked immune programming influence the threshold and downstream meaning (60). Such a framework may help explain why direct diet–itaconate associations are weak in free-living human populations, whereas sex-specific immune associations are more readily detected.

Some limitations should be acknowledged. The cross-sectional design precludes causal inference and does not allow us to determine whether changes in monocyte biology drive circulating itaconate, whether circulating itaconate shapes monocyte behavior, or both. Circulating itaconate was measured systemically and therefore cannot identify its tissue source or distinguish contributions from distinct myeloid compartments. Dietary exposure was assessed at the intake-pattern level and may not fully capture the complexity of diet quality, food matrix effects, or temporal variability. In addition, subgroup analyses by sex and legume category should be interpreted cautiously because they reduce effective sample size. Additionally, given the exploratory nature of the present analyses and the number of statistical comparisons performed, the possibility of type I error and false-positive findings cannot be excluded. Furthermore, sex-stratified analyses were performed in relatively small subgroups, which may have limited statistical power and should therefore be interpreted cautiously until replicated in larger cohorts. Finally, we did not measure sex hormones, macrophage polarization states, ACOD1 expression, or microbiome composition, all of which would help resolve mechanism. These limitations are balanced by relevant strengths, including the integration of dietary, inflammatory, anthropometric, and hematologic data, the explicit assessment of sex-specific associations, and the focus on a metabolite that is mechanistically anchored in myeloid immunometabolism. Future studies should move in three directions. First, longitudinal and intervention-based designs are needed to determine whether dietary modification, weight loss, or anti-inflammatory interventions alter circulating itaconate trajectories and whether these changes differ by sex. Second, mechanistic human studies should combine circulating metabolomics with monocyte/macrophage phenotyping, ACOD1 expression, ex vivo stimulation assays, and sex hormone profiling. Third, given the context-dependent effects of itaconate across cardiovascular disease, metabolic disease, inflammatory disorders, and cancer, future work should examine whether circulating itaconate can serve as a clinically useful biomarker only when interpreted together with immune-cell composition, inflammatory markers, and sex-stratified covariates.

## Conclusion

In conclusion, these findings support circulating itaconate as a biologically meaningful marker of innate immune-metabolic activation in humans, linked to monocyte-related phenotypes and sex-dependent in subjects with excessive body weight. Rather than being directly determined by legume intake where sex may have influences, circulating itaconate appears to reflect the interaction between inflammatory state, myeloid cell biology, metabolic milieu context, and sex-specific immune regulation. This framework may be particularly relevant for cardiovascular immunology, where low-grade inflammation, monocyte-macrophage dysfunction, and sex differences converge to shape disease susceptibility and progression with value for precision medicine and nutrition interventions.

## Data Availability

The datasets presented in this study can be found in online repositories. The names of the repository/repositories and accession number(s) can be found below: PRJNA1279632.
